# Research of the Influence of the Ultrasonic Treatment on the Melts of the Polymeric Compositions for the Creation of Packaging Materials with Antimicrobial Properties and Biodegrability

**DOI:** 10.3390/polym12020275

**Published:** 2020-01-30

**Authors:** Irina Kirsh, Yuliya Frolova, Olga Bannikova, Olga Beznaeva, Isabella Tveritnikova, Dmitry Myalenko, Valentina Romanova, Daria Zagrebina

**Affiliations:** 1 Scientific and Educational Center Advanced Packaging Materials and Recycling Technologies, Center of the Collective Use, Moscow State University of Food Production, 125080 Moscow, Russia; irina-kirsh@yandex.ru (I.K.); olgazaikina@mail.ru (O.B.);; 2 Laboratory of Food Biotechnology and Specialized Products, Federal Research Center of Nutrition and Biotechnology, 109240 Moscow, Russia; 3 Russian Research Institute of Dairy Industry, 115093 Moscow, Russia

**Keywords:** food packaging, antimicrobial properties, polyethylene, birch bark extract, ultrasound, thermoplastic starch, biodegradation

## Abstract

Ensuring the microbiological safety of food products is a problem of current interest. The use of antimicrobial packaging materials is a way of solving the problem. When developing packaging materials, it is advisable to use a modern approach based on the creation of biodegradable materials. The difficulty in the selection of the polymer compositions’ components lies in solving the dilemma of the joint introduction and processing of antimicrobial and biodegradable agents. The studies of the ultrasound treatment on the melts of polymer mixtures showed an increase in the dispersion process of the components of the mixture. In this regard, this work aimed to study the effect of the ultrasonic treatment on the melts of polymer compositions containing thermoplastic starch and birch bark extract (BBE). In the work, the properties of PE-based packaging materials with various BBE concentrations obtained with ultrasonic treatment of melts on a laboratory extruder were studied. Biodegradable polymer compositions containing thermoplastic starch and BBE, obtained with the use of the ultrasonic treatment during extrusion, were investigated. The methods for studying rheological, physic-mechanical, antimicrobial properties and sanitary chemical indicators of materials were used in the article. It was found that ultrasonic treatment increases the melt flow and contributes to the production of materials with the uniform distribution of additives. The BBE content from 1.0% and higher in the contents of the material provides antimicrobial properties. When studying the permeability of oxygen and water vapor of the polymer compositions based on PE and BBE, it was found that the introduction of a filler increases vapor permeability by about 8–12% compared with control samples. The optimal concentration of BBE in polyethylene compositions containing thermoplastic starch was determined. The extension of the shelf life of the food product during storage in the developed material was established.

## 1. Introduction

Ensuring the quality and safety of food products is one of main problems in the food industry [1]. Losses of unpackaged food products associated with spoilage can reach up to 50% [2]. Improving production and processing technologies, as well as correctly selecting packaging material, can significantly reduce product losses [1]. The safety of food products, of both animal and plant origin, is determined primarily by microbiological indicators, taking into account the quantitative and qualitative content of contaminants of chemical, biological and microbiological nature [3]. Loss of food integrity is the result of chemical and microbiological spoilage, which are the main causes of spoilage of many products during production, transportation, processing, storage and marketing. These processes are directly connected with the loss of the food products’ quality [1,4]. According to statistics, 80% of food poisoning is due to the presence of sanitary-indicative, pathogenic microorganisms, yeast, molds and their toxins in the products. When microorganisms get to the surface of a food product, due to the favorable environment, they develop, worsening the appearance, reducing taste, chemically reacting with the components, causing change in proteins and lipids, producing toxic substances that cause food poisoning and also creating favorable conditions for the growth of bacteria [5,6]. In this regard, one of the current trends in the food industry is the creation of packaging materials with antimicrobial properties.

Over the past decades, many packaging materials based on polymers and coatings with antimicrobial properties have been developed [1,7,8,9,10,11,12]. Substances of natural [8,13,14] and synthetic [15,16] origin, including nanoparticles, are used as antimicrobial agents [12,17]. Polyolefin-based materials (polyethylene, polypropylene) are often used as barrier packaging. However, polyethylene and polypropylene films do not have inherent antimicrobial properties [12]. Various approaches are used to impart antimicrobial properties to polymeric materials based on polyolefins: surface treatment of the polymeric material with antimicrobial additives (spraying onto the surface) followed by fixing the antimicrobial agent on the packaging surface [12] or the introduction of antimicrobial components directly into the polymer matrix [13,15,16,18,19,20]. Using the approach of the surface treatment of packaging materials with antimicrobial agents has a significant drawback in that there is a risk of the migration of the antimicrobial component into the food product, which can change its organoleptic characteristics. The advantage of this method is the possibility of using thermally unstable antimicrobial additives. When using this type of packaging material, it is necessary to strictly control the concentration and possible conditions of the migration of the antimicrobial additive into the product. Obtaining packaging materials using the second approach consists of directly mixing the additive and the polymer before loading into the extruder, followed by obtaining the material. The disadvantage of this method is the possibility of using only heat-resistant antimicrobial additives. Also, when mixing the additive and the polymer before loading into the extruder, there is the possibility of uneven distribution of the antimicrobial additive in the polymer matrix of the finished material. 

A promising antimicrobial additive, able to modify the properties of polyolefins, is birch bark extract (BBE). BBE (C_36_H_60_O_3_) is the multicomponent mixture containing betulinol, lupeol, lupenon, uveol, betulinol acetate, allobetulin, isobetulinol, oleanolic acid and other substances [21].

BBE is known for its antibacterial, antiviral, anti-inflammatory and antimutagenic properties, as well as its resistance to molds and bacteria [21,22]. BBE is resistant to oxygen and sunlight and is nontoxic, which allows it to be used in polymeric materials in contact with food. To intensify the production process of polymeric materials with a given set of properties, including those with antimicrobial properties, it is advisable to use superconcentrates (or masterbatches) [23,24]. This technology involves obtaining packaging material with a modifier in two stages: obtaining superconcentrate, followed by mixing it with a polymer base and obtaining a film. This approach allows obtaining polymer materials with the more uniform distribution of additives in the polymer matrix. However, at high concentrations of antimicrobial additives, the probability of its agglomeration increases and, as a consequence, the uneven distribution is possible. The formation of structures with the uniform distribution of component composition can be achieved by additional mechanical mixing, the use of dispersants, etc. However, the use of ultrasonic treatment of polymer melts is gaining practical and scientific interest [25,26]. The work performed to study the effect of the ultrasound treatment on the melts of polymer compositions containing agricultural wastes showed the increase in the uniform distribution of the mixture components even when the filler content is greater than 30%. In addition, it was found that ultrasonic treatment of melts of polymer compositions leads to the acceleration of the destruction of the polymer matrix due to the increase in oxygen-containing groups in polyethylene and the increase in water absorption [27]. Therefore, to improve the dispersion process of the components of polymer compositions, a laboratory extruder with ultrasonic treatment of polymer melts, developed at the university, was used. The aim of the present work was to study the effect of the ultrasonic treatment of melts of polymer compositions in order to create packaging materials with antimicrobial properties that ensure prolonged shelf life of packaged food products and have the ability to accelerate biodegradation.

## 2. Materials and Methods

### 2.1. Materials

In the experimental work birch bark extract (BBE) (Birch World, St. Petersburg, Russia), which is the multicomponent mixture containing betulinol, lupeol, lupenon, uveol and other substances, was used. It is registered as a biologically active additive in the state register (certificate No. 77.99.23.3.U.3440.4.08 of April 29, 2008). Other materials used were high-pressure polyethylene (PE) (Kazanorgsintez, Kazan, Russia), grade Kazpelen 15813-020, the main characteristics of which are presented in Table 1; thermostabilizer Irganox 1010 (pentaerythritol tetrakis(3-(3,5-di-tert-butyl-4-hydroxyphenyl)propionate)) (BASF, The Chemical Company, Basel, Switzerland); corn starch with a particle size of 50 μm and a share of fraction up to 30 μm of at least 50%. Nutrient media for the microbiological research was as follows: dry nutrient medium Sabouraud (GNTsPMB, Obolensk Russia), dry nutrient medium Czapek-Dox (GNTsPMB, Obolensk, Russia), meat-peptone agar (GNTsPMB, Obolensk, Russia). All other reagents used to analyze the properties of the materials were of analytical purity.

### 2.2. The Technology of Polymer Materials

Polymer compositions based on polyethylene, thermoplastic starch and birch bark extract (BBE) were used. Thermoplastic starch was obtained in a laboratory mixer by mixing starch, plasticizers and additives to improve processing of the compositions. In polymer composite materials (PCM), the amounts of thermoplastic starch were 20%, 40% and 60%. The amount of BBE in PCM ranged from 1% to 12%.

Obtaining samples of films based on PE with BBE was carried out in two stages:

1. Obtaining PE with BBE in the form of granules with diameter 3 mm and length 6 mm.

2. Obtaining film material (film thickness 62 ± 2 μm) from granules of PE with BBE and thermoplastic starch.

To obtain granules and film material, the laboratory extruder with the screw diameter of 16 mm and with ultrasonic (US) treatment of the melt, developed at the university, was used. Ultrasonic treatment of the melt of the polymer compositions was conducted at 22.4 kHz. Processing modes are presented in Table 2.

The use of the ultrasonic treatment allowed the production of materials with the temperatures of extruder zones 3 and 4 being 10 °C lower than without the ultrasonic treatment.

The concentration of BBE in PE varied between 0.5%, 1.0%, 2.0% and 5.0%. Irganox 1010 (BASF, The Chemical Company, Basel, Switzerland) was introduced as thermostabilizer in the amount of 1.0%. The content of the compositions is presented in Table 3.

Materials, based on PE and BBE without ultrasonic treatment and pure PE, not containing BBE, were used as control samples. The concentration of BBE in PE with thermoplastic starch varied between 0%, 2.0%, 5.0%, 8.0% and 12.0%. Irganox 1010 was introduced as thermostabilizer in the amount of 1%. The amounts of thermoplastic starch were 20%, 40% and 60%. The content of the compositions is presented in Table 4.

Compositions obtained without the influence of ultrasound on their melts were selected as control samples.

### 2.3. Characteristic of Polymer Materials

#### 2.3.1. Appearance and Microstructure

The visual assessment of the developed films was carried out in order to determine the color of the outer and inner surfaces. The assessment of the surface of the sample aimed to detect cracks, sagging, bumps, roughness. Microphotographs of the surface were obtained using the Bresser digital light microscope (Bresser GmbH, Germany). The structural-morphological properties of the samples were studied using the scanning electronic microscope, JSM-U3 (JEOL, Tokyo, Japan).

#### 2.3.2. Rheological Research

Studies of polymer melts were carried out by the standard method of capillary viscometry [28]. This experiment was carried out five times.

#### 2.3.3. Determination of Physico-Mechanical Properties

Studies of polymeric materials samples were carried out on a universal testing machine BM-50 (Moscow, Russia) with fixation of and elongation at break. The tensile speed of the materials was 10 mm/s. This experiment was carried out five times.

#### 2.3.4. Determination of Antimicrobial Properties

Determination of Antibacterial Properties: To determine the antibacterial properties of polymeric materials the culture of microorganisms *Escherichia coli* M 17 (*E. coli*) and *Candida albicans* (*C. albicans*) was used. The microorganism culture was grown in 50 mL glass tubes with beveled nutrient agar for 24 h. As the nutrient medium, meat-peptone agar was used. The grown culture was suspended in physiological saline with the turbidity of 0.5 according to the McFarland standard (1.5 × 10^8^ CFU/mL) and was used for 15 min. Then, 22 ± 1 mL of meat-peptone agar was poured into Petri dishes and left until the agar solidified. Inoculation of the culture on the surface of the agar was carried out by distributing 0.2 mL of the suspension with a glass microbiological spatula of Drigalski. Next, disks of polymer material with the diameter of 20 mm were laid on the inoculated surface. Preliminarily, the polymeric material was disinfected by the double immersion in pure ethanol and the solvent vapor was allowed to evaporate completely before use. Petri dishes with the studied samples were placed in the TS-1/20 SPU thermostat (Smolensk SKTB SPU, Smolensk, Russia) at the temperature of 37 ± 1 °C for 48 h. After 24 h, the intermediate inspection of the plates was performed. The development of microorganisms on the materials’ surface and the presence of the inhibition growth zone were visually assessed. The experiment was conducted three times.

Determination of Fungicidal Properties: To determine the resistance of polymeric materials to mold, the following cultures were used: *Penicillium commune* F-4486 (*P. commune*) and *Aspergillus niger* strain 82 (*A. niger*). The studies were carried out in two ways. According to the first method, *P. commune* mold was grown in 50 mL glass tubes with beveled nutrient agar for 7 days. Sabouraud was used as the nutrient medium. The grown culture was suspended in physiological saline. The spore concentration in the solution was (2 ± 1) × 10^6^ CFU/mL. Then, the experiment was carried out according to the method described in Section 2.3.4. Petri dishes with the studied samples were placed in the TS-1/20 SPU thermostat (Russia) at the temperature of 28 ± 1 °C for 72 h. After 48 h, the intermediate inspection of the plates was performed. The development of microorganisms on the materials’ surface and the presence of the inhibition growth zone were visually assessed. The experiment was carried out three times. The second method consisted of keeping the polymer materials contaminated with the spores of *A. niger* mold in the absence of mineral and organic pollutants at high humidity. A fungus culture of *A. niger* was grown in test tubes with the beveled nutrient medium (Czapek-Dox medium with agar) for 14 days. The spore suspension was prepared in sterile distilled water. The spore concentration was (1.5 ± 0.5) × 10^6^ CFU/mL. Disks of polymer material with the diameter of 20 mm were cleaned before use according to the method described in Section 2.3.4. Prepared film samples were placed one per Petri dish. Inoculation of the samples was carried out by spraying the suspension of the mold spores on the surface of the film, preventing droplets from merging. The inoculated samples were kept in a box for the drying of drops at the temperature of 23 ± 1 °C for no more than 60 min. Petri dishes were closed and placed in desiccators, on the bottom of which was poured distilled water to create conditions of high humidity (greater than 90%). The desiccators were closed and kept in a dark room at the temperature of 29 ± 1 °C. The test duration was 14 days. After 7 days, the covers of the desiccators were opened for 3 min for the oxygen access. The surface was evaluated after incubation by light microscopy at the magnifications of 100× and 400×. The experiment was carried out three times.

#### 2.3.5. Sanitary Chemical Research

The organoleptic evaluation was performed in the universal model medium (distilled water). The purified polymeric material was immersed in the model medium in the ratio 1:1 and kept at temperatures of 20, 40 and 60 °C for 7, 14, 21 and 28 days. During the organoleptic assessment, the presence of turbidity, sediment and extraneous odor of aqueous extracts was determined. The turbidity of the extracts was characterized descriptively as either lack of turbidity, weak opalescence, opalescence, strong opalescence, weak turbidity, noticeable turbidity or strong turbidity. Sediment was characterized by the absence of sediment as either negligible, insignificant, noticeable and large. Its properties were noted as crystalline, amorphous, etc. Its color was also noted. The smell of water extracts was expressed descriptively as lacking smell, phenolic, aromatic, extraneous indefinite, etc. Odor intensity was expressed on a 5-point scale, where 0 indicated no tangible smell and 5 indicated an obviously strong smell that caused a persistent negative feeling. The experiment was carried out three times.

Quantitative assessment of the migration of low-molecular-weight substances from polymeric materials to model media (2.0% citric acid; 3.0% lactic acid; 0.3% solution of sodium chloride) was carried out by the standard gas chromatographic method with the use of high-performance modular liquid chromatography (Agilent 1200, Agilent Technologies Inc, USA).

#### 2.3.6. Determination of Permeability of Packaging Materials

To determine the vapor permeability of the packaging materials, the method of determining the amount of water vapor through polymeric materials by changing the mass of the container with distilled water and a sample over 24 h at the temperatures of 23 and 38 °C and the humidity of 90–98% (ASTM D1653) was used [29]. The appliance W3/030 Labthink (Labthink Instruments Co. Ltd., China (CPR)) was used.

To determine oxygen permeability of the packaging materials, the equilibrium pressure method at the temperature 23 °C (GB/T 1038-2000) was used [30]. The appliance PERME OX2/231 Labthink (Labthink Instruments Co. Ltd., China (CPR)) was used.

#### 2.3.7. Biodegradability Assessment of the Materials

The samples were placed in containers with biohumus at the temperature 23 ± 2 °C and the humidity 60% ± 5% [31]. Samples of polymeric materials were cut out with a size of 10 × 10 cm and placed in a container with soil at a humidity of at least 50% of its maximum moisture capacity. A layer of soil 1.5 ± 0.5 cm thick was poured on top of the samples and loosely closed containers were placed in a chamber at a temperature of 23 ± 2 °C and humidity of 60% ± 5%. The temperature and humidity levels were monitored throughout the composting process. Composting time for polymeric materials was 6 months. The degree of biodegradation of the materials was determined by the change in physico-chemical properties during composting.

### 2.4. Packed Product Storage Studies

Studies of the storage of products in polymeric materials were carried out on chilled parts of broiler carcasses. Fresh carcass parts were bought in a local supermarket. The weight of each part of the carcass was 120 ± 30 g. Fresh carcass parts were hermetically sealed in the polymeric material under sterile conditions. The control samples were carcass parts of the same batch, packaged under the same conditions in PE without BBE. The packaged products were stored for 5 days at the temperatures from 0 to 2 °C. The content of the quantity of mesophilic aerobic and facultative anaerobic microorganisms in the product were controlled as a standardized indicator of microbiological spoilage. Control points were 0, 2, 3, 4 and 5 days. ISO 4833:2003 conventional method for determining the quantity of mesophilic aerobic and facultative anaerobic microorganisms (QMAFAnM) was used [32].

To determine the optimal concentration of BBE in PE compositions containing thermoplastic starch, the relative increase in shelf life was used as a criterion, which was calculated by the following formula:

(1) $$W=\frac{ti-tk}{tk}\times 100\%$$

where *W* is the relative increase in shelf life; *ti* is product storage time in PCM-based films; *tk* is the storage time in films based on PE without additives.

### 2.5. Statistics

Statistical processing of the results was performed using the IBM SPSS Statistics Program 20 (SPSS Inc. USA).

## 3. Results and Discussion

### 3.1. Study of the Effect of the Ultrasonic Treatment on the Properties of Polyethylene Compositions Modified by BBE

#### 3.1.1. Appearance

**Figure 1:**
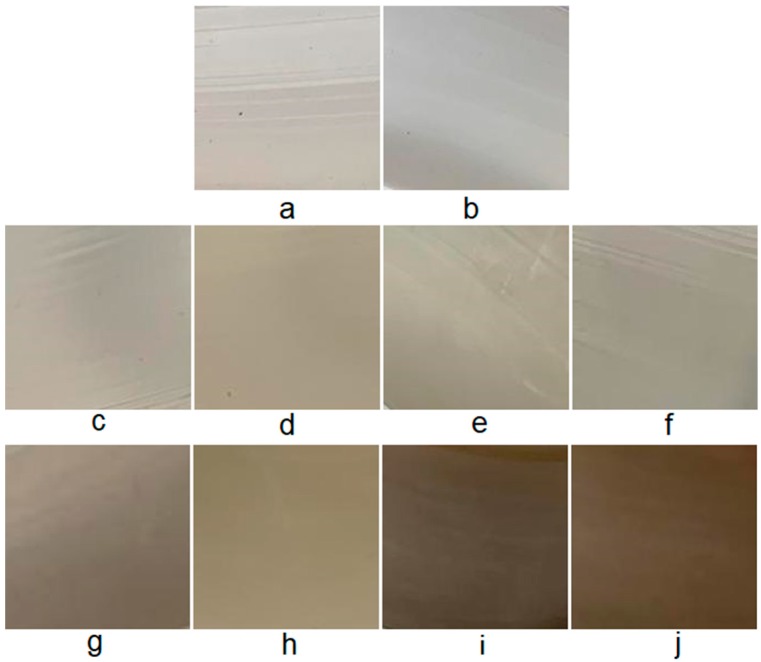
External appearance of the obtained polymeric materials, containing 0%, 0.5%, 1.0%, 2.0% and 5.0% of birch bark extract (BBE) and received with the use of ultrasonic treatment (with US) and without the use of ultrasonic treatment (without US): (**a**) 0% BBE without US; (**b**) 0% BBE with US; (**c**) 0.5% BBE without US; (**d**) 0.5% BBE with US; (**e**) 1.0% BBE without US; (**f**) 1.0% BBE with US; (**g**) 2.0% BBE without US; (**h**) 2.0% BBE with US; (**i**) 5.0% BBE without US; (**j**) 5.0% BBE with US.

The appearance of the obtained polymer films is presented in Figure 1.

**Figure 2:**
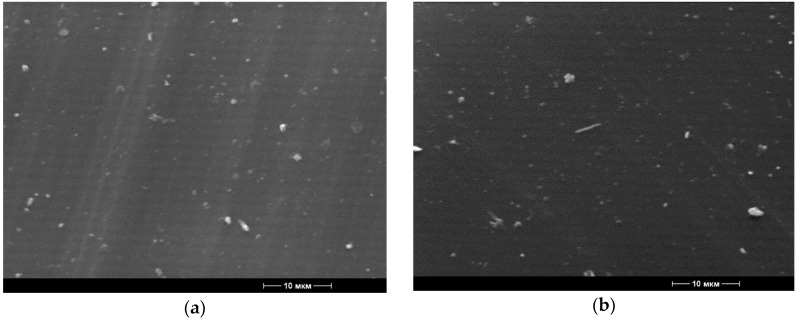
Microphotographs of the surface structure of the materials (5000× magnification) based on polyethylene with 2.0% content of birch bark extract, received with the use of ultrasonic treatment (**a**) and without the use of ultrasonic treatment (**b**).

**Figure 3:**
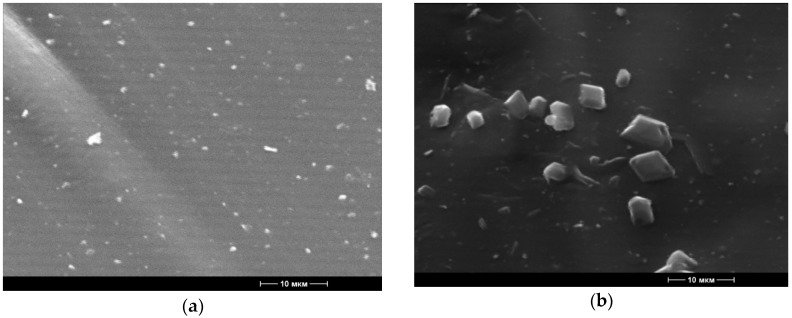
Microphotographs of the surface structure of the materials (5000× magnification) based on polyethylene with 5.0% content of birch bark extract, received with the use of ultrasonic treatment (**a**) and without the use of ultrasonic treatment (**b**).

The inclusion of BBE in the polymer matrix of polyethylene leads to the color change of the materials obtained. At low concentrations (1–2% BBE), the films had slight yellowness (Figure 1e–h), and at high concentrations (5% BBE), the films turned brown (Figure 1i–j). The films containing BBE had a rough but smooth surface. The samples of films obtained without the treatment of ultrasonic melt were distinguished by the presence of the agglomerated filler; with the increase of the BBE content, the number of agglomerates increased. The agglomerate formation was confirmed by electron microscopy images (Figure 2 and Figure 3).

The use of the ultrasonic treatment of the polymer melt in the production of materials with BBE made it possible to obtain more transparent samples with the more uniform distribution of the additive in the material.

#### 3.1.2. Rheological Properties

**Figure 4:**
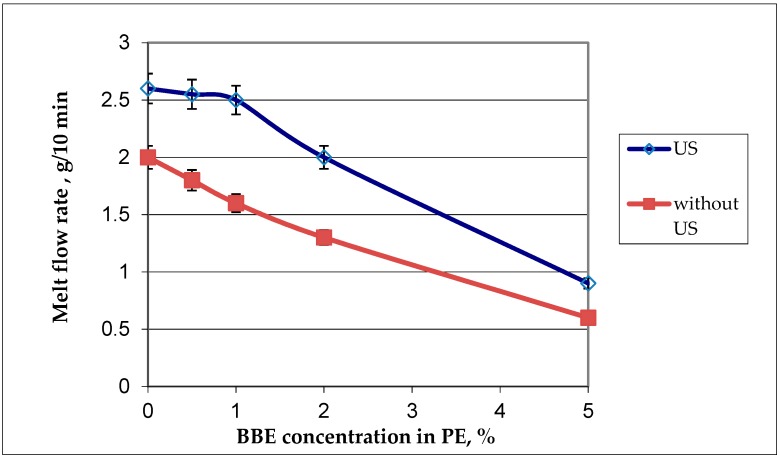
Graphic dependance of the melt flow rate on the birch bark extract (BBE) content in polyethylene (PE) and on ulrasonic (US) treatment.

Figure 4 shows the dependence of the melt flow rate on the concentration of BBE and ultrasound.

Processing of ultrasonic melts leads to the increase in the MFR of pure polyethylene and compositions with BBE. The increase in the MFR value under the influence of the ultrasonic treatment is associated with the peculiarities of the effect of the ultrasonic treatment on polymer melts, as presented in [25]. The increase in the content of BBE in the composition of PE leads to the decrease in the MFR, this is due to the fact that the BBE introduced into the polymer behaves as the “filler-polymer”.

#### 3.1.3. Physico-Mechanical Properties

**Figure 5:**
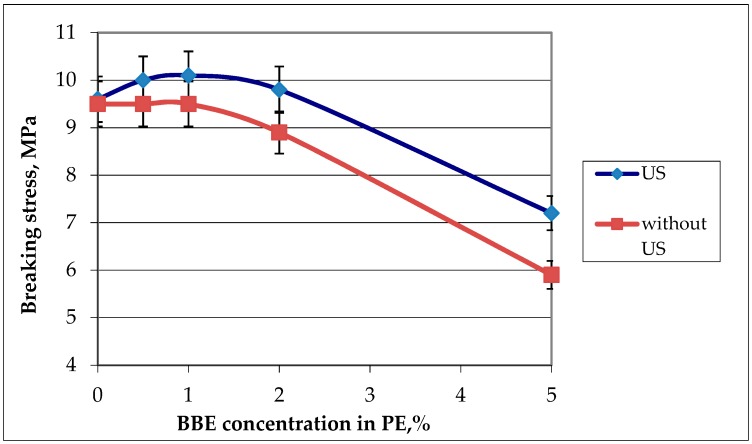
Graphic dependence of the breaking stress on the birch bark extract (BBE) content in polyethylene (PE) and on ultrasonic (US) treatment.

**Figure 6:**
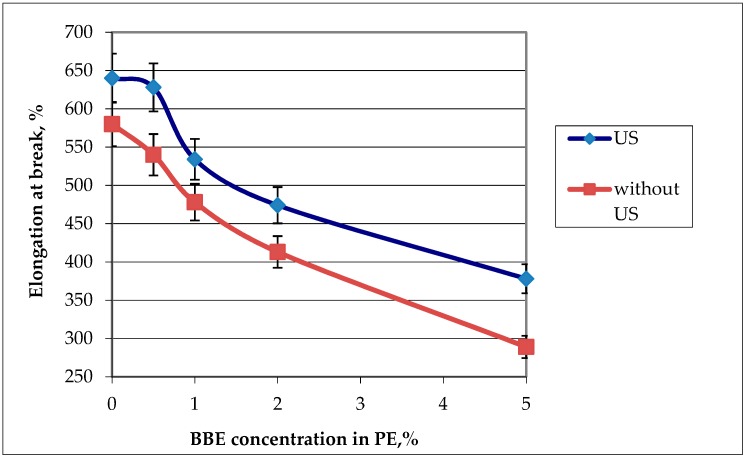
Graphic dependence of the elongation at break on the birch bark extract (BBE) content in polyethylene (PE) and on ultrasonic (US) treatment.

The influence of the BBE concentration and ultrasound treatment on the physico-mechanical properties was evaluated by two indicators: breaking stress and elongation at break. The results are presented in Figure 5 and Figure 6.

It was found that with the increase of the concentration of BBE in the composition of the polymeric material based on PE, the breaking stress and elongation at break also increase, which is connected with the distribution of the BBE in the structure of PE and the violation of the integrity of its primary structure. Ultrasonic treatment of melts in the production of polymeric materials leads to the increase in breaking stress and elongation at break by 1.5 times in comparison with the control samples. This is due to the fact that, during the ultrasonic treatment, the distribution of the additive is uniform (Figure 1, Figure 2 and Figure 3). The low content of BBE (up to 1%) in the composition of the polymeric material has practically no effect on the physico-mechanical properties.

#### 3.1.4. Antimicrobial Properties

The results of the evaluation of the antimicrobial properties of the studied materials samples in relation to *E. coli*, *C. albicans* and *P. commune* are presented in Table 5.

**Figure 7:**
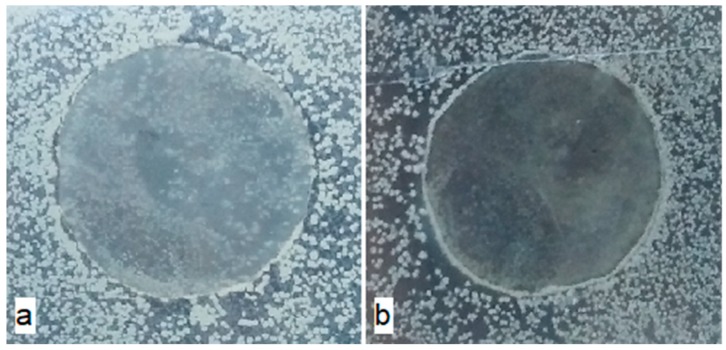
External appearance of polyethylene without birch bark extract (**a**) and polyethylene with 1.0% content of birch bark extract (**b**) and the use of ultrasonic treatment after the incubation under the influence of *C. albicans* for 24 h.

Based on the data obtained, it was found that ultrasonic treatment of melts during the development of materials does not affect their antimicrobial properties. The dependence of antimicrobial activity on the concentration of BBE in the polymer matrix was revealed. For *C. albicans*, after 24 h of exposure, the growth of cultures was observed on the surface of the control samples containing no BBE (Figure 7a).

The pattern of the antimicrobial properties of the materials with BBE compared with the control samples (example, Figure 7) was observed for all cultures of microorganisms: *E. coli*, *C. albicans* and *P. commune.* At the BBE concentration of 0.5% for 24 h, the growth inhibition was observed on the surface of the materials, but after 48 h there was the growth on the surface. The development of *P. commune* occurred with a certain delay compared to *E. coli and C. albicans*, which is connected with the peculiarities of the growth rate of microorganisms, in this regard, the development of molds was evaluated after 48 h. Materials containing 1% and 2% BBE are characterized by the absence of growth of microorganisms on their surface and under the material for 168 h of exposure. In this regard, materials containing 1% and 2% BBE have bacteriostatic and fungistatic properties. The increase in the BBE content to 5% leads to the appearance of the inhibition zone (3.0 ± 0.5 mm), which may indicate the migration of BBE into the agar medium.

**Figure 8:**
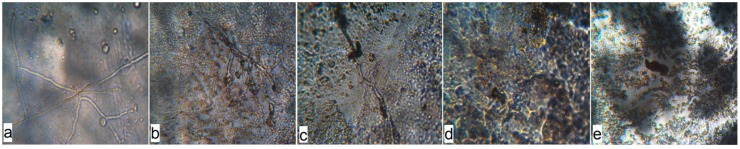
Microphotographs of the surface of the inoculated polyethylene materials with birch bark extract and with the use of ultrasonic treatment on the 14th day of *A. niger* exposure (100× magnification): (**a**) PE 0% BBE ultrasound; (**b**) PE 0.5% BBE, ultrasound; (**c**) PE 1.0% BBE, ultrasound; (**d**) PE 2.0% BBE, ultrasound; (**e**) PE 5.0% BBE, ultrasound.

In addition, the stability of materials with BBE exposure under conditions of high humidity (greater than 90%) at 29 ± 1 °C was evaluated against *A. niger* according to the degree of fungus development on the film surface, using a microscope (Figure 8).

**Figure 9:**
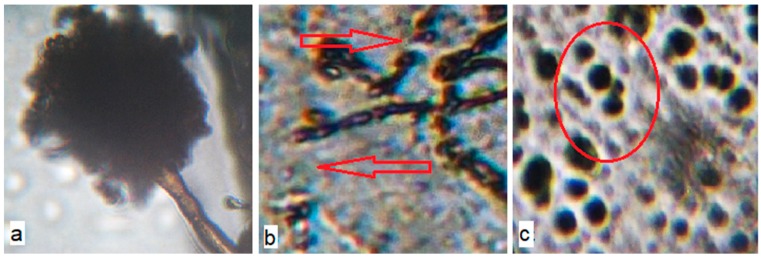
Microphotographs of the surface of the inoculated polyethylene (PE) materials with birch bark extract (BBE) and with the use of ultrasonic treatment on the 14th day of the exposure of *A. niger* (400× magnification): (**a**) PE 0% BBE, ultrasound; (**b**) PE 0.5% BBE, ultrasound; (**c**) PE 2.0% BBE, ultrasound.

On the surface of the materials based on PE without BBE, after 14 days of exposure under conditions of forced surface inoculation with *A. niger* fungus at a temperature of 29 ± 1 °C and humidity of greater than 90%, the formation of mycelium (Figure 8a) and conidia with conidiospores (Figure 9a) was observed. On the surface of PE materials with 0.5% BBE, partial destruction of mycelium was observed after 14 days of exposure (Figure 8b and Figure 9b), which indicates the death of the culture. On microphotographs of the surface of the material with BBE concentration of 2.0%, complete destruction of the mycelium was observed (Figure 8d and Figure 9c). Based on the results of the growth of *A. niger* culture on the surface of materials containing BBE, it was found that on the surface of materials containing 2.0% or more BBE, under conditions of forced inoculation, the culture is destroyed, which indicates the mold resistance of materials with BBE. On the basis of rheological, physico-mechanical and antimicrobial studies of materials based on PE with BBE obtained with and without ultrasonic treatment, it is recommended to use material containing 2.0% BBE for packaging to ensure microbiological safety of food products.

#### 3.1.5. Sanitary Chemical Research

Materials intended for contact with food products were subjected to sanitary chemical studies for the migration of low-molecular-weight substances.

Organoleptic studies at temperatures of 20, 40 and 60 °C for 7, 14, 21 and 28 days showed that, in the analyzed extracts from all the materials, there was no sediment, turbidity or color change in the extracts. However, there was the increase in the intensity of odor from materials with the increase in BBE concentration and temperature to 60 °C, while the average value was no more than 1 point, which is allowed for the materials contacting food products.

The results of the study of the migration of low-molecular-weight substances from the materials are presented in Table 6.

When using a model medium of 3.0% lactic acid solution, the increase in the migration of methyl alcohol and formaldehyde is observed, although its value does not exceed permissible norms (Table 6). A study of the migration of low-molecular-weight components from developed materials with BBE shows that migrating substances do not exceed permissible norms. It was found that ultrasonic treatment of the melt and the content of the additive at the concentration of up to 5% do not affect the formation of low-molecular-weight substances that can migrate into the food product upon contact. 

Based on the carried out rheological, physico-mechanical, antimicrobial and sanitary chemical studies, it is proposed to use 2% BBE with ultrasound treatment as the packaging material for contact with food products. This material has sufficient physico-mechanical properties, has antimicrobial properties and complies with sanitary chemical standards. The use of the ultrasound treatment makes it possible to obtain material with uniformly distributed additive, and the content of 2% BBE is justified by the economic factor.

#### 3.1.6. Study of the Permeability of the Packaging Materials

When studying the vapor permeability of polymeric compositions based on PE and BBE, it was found that the introduction of a filler increases the vapor permeability by about 8–12% in comparison with the control samples. For example, the vapor permeability of PE without BBE is 0.18–0.21 g/m^2^. When the BBE content is 5% in the polyethylene composition, the vapor permeability is about 0.23–0.25 g/m^2^.

When studying the oxygen permeability of the samples, it was found that oxygen permeability coefficients for the compositions based on PE and BBE 5% are 1.42 × 10^−10^ cm^3^ cm/m^2^ s Pa. For the PE film without BBE, the oxygen permeability coefficient is 1.2 × 10^−10^ cm^3^ cm/m^2^ s Pa.

### 3.2. Packed Product Storage Studies

The results of storing chilled meat are presented using broiler chicken carcasses’ parts as an example. Packed product samples were stored at 0 ± 2 °C. The quantity of mesophilic aerobic and facultative anaerobic microorganisms (QMAFAnM) was determined by the accelerated method of seeding on test plates—petrifilms containing dehydrated nutrient gel-like chromogenic substrate.

In the process of storage, the quantity of mesophilic aerobic and facultative anaerobic microorganisms increases in the packed parts of poultry carcasses (Table 7). The acceptable content of QMAFAnM in chilled poultry is 1 × 10^3^ CFU/g. It was found that during the storage of poultry carcasses at the temperature of 0 ± 2 °C for 4 days in a packaging material based on pure polyethylene (PE), the QMAFAnM index approaches a critical value and significantly exceeds it by 5 days. The use of such products for food is considered unsafe for health. During the storage of poultry carcasses at the temperature of 0 ± 2 °C, the QMAFAnM index approaches the critical value on the 5th day. The rate of accumulation of the quantity of mesophilic aerobic and facultative anaerobic microorganisms is higher in a product packaged in pure polyethylene compared to a product packaged in plastic film with birch bark extract. This is due to the fact that upon the contact of the packaging material containing BBE with the product, partial suppression of the growth of microorganisms located on its surface occurs.

The data obtained show that the use of the developed PE BBE material in the packaging technology of chilled meat (poultry meat in particular) leads to the increase in shelf life.

### 3.3. Investigation of the Effect of Ultrasonic Treatment on the Properties of Polyethylene Compositions Based on Birch Bark Extract and Thermoplastic Starch

At this stage of the work, the development of polymer composite materials (PCM) with antimicrobial properties and biodegradability was carried out. In this work, compositions based on PE, thermoplastic starch and BBE were obtained. In PCM, the amounts of thermoplastic starch were 20%, 40% and 60%. The amount of BBE in PCM ranged from 0% to 12%.

The obtained film samples were investigated by rheological and physico-mechanical properties (Table 8 and Table 9).

From the results obtained, it is clearly seen that the ultrasonic treatment increases the melt flow rate by approximately 2 times compared with the control samples obtained without ultrasonic treatment.

**Figure 10:**
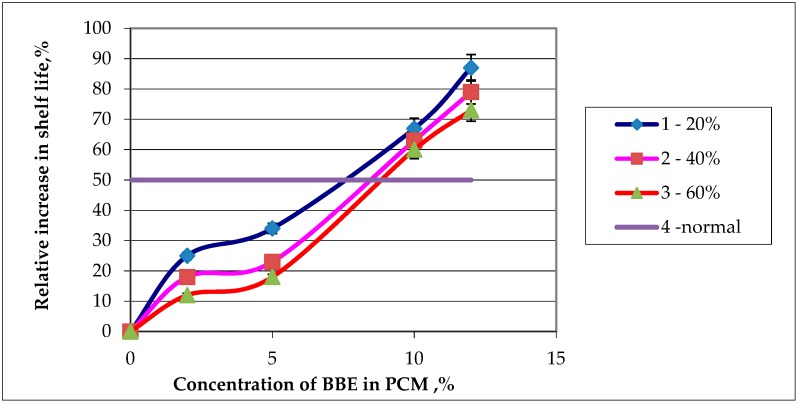
Dependence of the relative increase in shelf life of the food product on the contents of birch bark extract (BBE) in composition material (PCM): (1—content of thermoplastic starch 20% in PCM; 2—content of thermoplastic starch 30% in PCM; 3—content of thermoplastic starch 60% in PCM; 4—desired value to increase product storage).

It is clearly seen that the introduction of up to 5% BBE has little effect on the physico-mechanical properties of PCM. The introduction of thermoplastic starch is of great importance; its amount reduces the breaking stress and elongation at break. It has been established that the processing of PCM melts increases the physico-mechanical properties of the materials, which is especially noticeable when comparing the elongation at break. The values of this indicator are approximately 1.5–2 times greater for PCMs obtained with ultrasonic treatment of the melt as compared to control samples. To determine the optimal concentration of BBE in PCM, studies were conducted to determine the shelf life of food products in packages. Dependence of the relative increase in shelf life on the concentration of BBE in polymer compositions containing thermoplastic starch is shown in Figure 10.

Based on the data obtained, BBE concentration of 8% was selected. 

Next, the research was conducted using the PCM method of composting (Table 10). As the criterion for evaluating the properties, the change in elongation at break after 6 months of composting was used.

It is worth noting that the samples obtained with ultrasonic treatment showed more noticeable change in relative elongation at break after composting than those without ultrasonic treatment. This indicates that the effect of ultrasound treatment accelerates the decomposition of polymer materials in the environment due to the uniform distribution of the filler in the polymer matrix and the increase in oxygen-containing groups in polymers, which was noted in the [27]. It should be noted that the introduction of BBE in PCM leads to the decrease in this indicator. This can probably be attributed to the action of BBE as the antimicrobial supplement.

Special attention should be paid to the material based on PE containing 60% starch and 8% BBE, in which, after 6 months of composting, the elongation at break changed by 60–68%. This is a good criterion for obtaining biodegradable PCMs.

## 4. Conclusions

The influence of ultrasonic treatment of melts of the polymer compositions based on PE and BBE, as well as PE, thermoplastic starch and BBE, was investigated. Packaging materials in the form of films based on polymer compositions were obtained. A comparison of identical PCM compositions obtained with and without ultrasonic treatment of the melts revealed that the ultrasonic treatment increases the fluidity of the melts of polymer compositions. Using electron microscopy, it was found that ultrasonic treatment of the melts of polymer compositions contributes to the production of materials with the uniform distribution of the components of the composition, as shown by the example of a PE sample containing 2% and 5% BBE. When studying oxygen and water vapor permeability of polymeric compositions based on PE and BBE, it was found that the introduction of the filler increases vapor permeability and oxygen permeability by about 8–12% compared to control samples without the addition of BBE. Ultrasonic treatment of polymer compositions does not affect these indicators. It was established that the processing of PCM melts increases the physico-mechanical properties of the materials, which is especially noticeable when comparing the elongation at break. The values of this indicator are approximately 1.5–2 times higher for PCMs obtained with ultrasonic treatment of the melt as compared to control samples. It is predetermined that the BBE content of 1.0% and above in the composition of PE provides packaging materials with antimicrobial properties. The optimal concentration of BBE in compositions based on PE and thermoplastic starch was determined. To obtain biodegradable materials with antimicrobial properties based on PE and thermoplastic starch, it is advisable to introduce BBE in the amount of greater than 8%. It was found that the extension of the shelf life of the food products stored in packaging materials based on PE and BBE in the amount of 2.0% and PE containing thermoplastic starch (60%) and BBE (8%), was greater than 50%, when compared with the control samples without additives. It was revealed that ultrasonic treatment of polymer compositions leads to the acceleration of their biodegradation. The introduction of BBE into PCM leads to the decrease in the biodegradation time, which is associated with the action of BBE as the antimicrobial supplement.

## Figures and Tables

**Table 1:** The main characteristics of polyethylene.

Parameter	Value
Average molecular weight	78,600
Melting point, mp, °C	105–108
Density, ρ, g/cm^3^, ρ	0.919–0.925
Melt flow rate, g/10 min	2–2.5
Elongation at break, ε_p_, %	600–800
Breaking stress, σp, MPa	11–15

**Table 2:** Temperature conditions for the processing of the polyethylene compositions in the extruder.

Temperature in the Extruder by Zone, °C				Polymer Compositions ^2^
Zone 1 ^1^	Zone 2	Zone 3	Zone 4	
120	160	180	190	PE with BBE
120	150	150	160	PE with BBE and starch

^1^ Zones 1–3 represent zones in the extruder; zone 4 is the extrusion head. The ultrasonic processing frequency was 22.4 kHz, the ultrasonic power was 300 W, and the screw rotational speed was 90 rpm. ^2^ PE with BBE, polymeric composition based on polyethylene with birch bark extract; PE with BBE and starch, polymeric composition based on polyethylene with birch bark extract and thermoplastic starch.

**Table 3:** Component composition of polymeric compositions based on polyethylene (PE) with birch bark extract (BBE).

Polymer Composition	The Content of the Compositions, %		
	Polyethylene	Birch Bark Extract	Irganox 1010
PE 0.5% BBE	98.5	0.5	1
PE 1.0% BBE	98	1	1
PE 2.0% BBE	97	2	1
PE 5.0% BBE	94	5	1
PE 0% BBE	100	0	0

**Table 4:** The component composition of the polymeric compositions based on polyethylene (PE) with birch bark extract (BBE) and thermoplastic starch.

Polymer Composition	The Content of the Compositions, %		
	Polyethylene with Irganox 1010	Birch Bark Extract	Thermoplastic Starch
PC 20 BBE 0	80	0	20
PC 40 BBE 0	60	0	40
PC 60 BBE 0	40	0	60
PC 20 BBE 2	78	2	20
PC 40 BBE 2	58	2	40
PC 60 BBE 2	38	2	60
PC 20 BBE 5	75	5	20
PC 40 BBE 5	55	5	40
PC 60 BBE 5	35	5	60
PC 20 BBE 8	72	8	20
PC 40 BBE 8	52	8	40
PC 60 BBE 8	32	8	60
PC 20 BBE 12	68	12	20
PC 40 BBE 12	48	12	40
PC 60 BBE 12	28	12	60

**Table 5:** Results of the visual assessment of the surface of polymeric materials based on polyethylene (PE) with birch bark extract (BBE) received with (with US) and without the use of ultrasonic (without US) treatment, inoculated for 24–48 h.

Polymer Composition	Visual Assessment		
	*E. coli*	*C. albicans*	*P. commune*
PE 0% BBE without US	Surface growth	Surface growth	Surface growth
PE 0% BBE with US	Surface growth	Surface growth	Surface growth
PE 0.5% BBE without US	Slow surface growth	Slow surface growth	Slow surface growth
PE 0.5% BBE with US	Slow surface growth	Slow surface growth	Slow surface growth
PE 1.0% BBE without US	Lack of surface growth	Lack of surface growth	Lack of surface growth
PE 1.0% BBE with US	Lack of surface growth	Lack of surface growth	Lack of surface growth
PE 2.0% BBE without US	Lack of surface growth	Lack of surface growth	Lack of surface growth
PE 2.0% BBE with US	Lack of surface growth	Lack of surface growth	Lack of surface growth
PE 5.0% BBE without US	Zone of inhibition 1.8 ± 0.2 mm	Zone of inhibition 1.5 ± 0.1 mm	Zone of inhibition 3.0 ± 0.5 mm
PE 5.0% BBE with US	Zone of inhibition 1.7 ± 0.2 mm	Zone of inhibition 1.5 ± 0.2 mm	Zone of inhibition 3.1 ± 0.5 mm

**Table 6:** Results of sanitary chemical research of extarcts of various model environments of materials based on polyethylene with 2.0% content of birch bark extract and the use of ultrasonic treatment.

Name of Indicator, mg/dm^3^	Permissible Norm	Citric Acid 2.0%	Lactic Acid 3.0%	Solution NaCl 0.3%
Acetaldehyde	≤0.2	<0.05	<0.05	<0.05
Ethyl acetate	≤0.1	<0.05	<0.05	<0.05
Hexane	≤0.1	<0.05	<0.05	<0.05
Heptane	≤0.1	<0.05	<0.05	<0.05
Acetone	≤0.1	<0.05	<0.05	<0.05
Formaldehyde	≤0.1	<0.025	<0.025	<0.025
Methyl alcohol	≤0.2	0.08	0.12	0.17
Butyl alcohol	≤0.5	<0.05	<0.05	<0.05
Isobutyl alcohol	≤0.5	<0.05	<0.05	<0.05
Propyl alcohol	≤0.1	<0.05	<0.05	<0.05
Isopropyl alcohol	≤0.1	<0.05	<0.05	<0.05

**Table 7:** Change in the content of the quantity of mesophilic aerobic and facultative anaerobic microorganisms, CFU/g, in parts of poultry carcasses during storage.

Type of Packaging	Duration of Storage, days				
	0	2	3	4	5
PE 0% BBE US	<10^2^	(5.75 ± 0.30) × 10^2^	(7.40 ± 0.28) × 10^2^	(0.95 ± 0.20) × 10^3^	(2.11 ± 0.14) × 10^3^
PE 2% BBE US	<10^2^	(3.10 ± 0.22) × 10^2^	(5.50 ± 0.20) × 10^2^	(0.72 ± 0.18) × 10^3^	(0.90 ± 0.21) × 10^3^

**Table 8:** Dependance of the melt flow rate index (MRF) on the composition of polymeric compositions material, received with (with US) and without the use of ultrasonic treatment (without US).

Polymer Composition	Average Values MRF, g/10 min	
	with US	without US
PC 20 BBE 0	1.7	1.2
PC 40 BBE 0	1.2	0.7
PC 60 BBE 0	0.9	0.6
PC 20 BBE 2	1.3	1.0
PC 40 BBE 2	0.9	0.8
PC 60 BBE 2	0.7	0.4
PC 20 BBE 5	1.1	0.8
PC 40 BBE 5	0.8	0.4
PC 60 BBE 5	0.5	0.2
PC 20 BBE 8	0.9	0.5
PC 40 BBE 8	0.4	0.2
PC 60 BBE 8	0.3	0.1
PC 20 BBE 12	0.8	0.5
PC 40 BBE 12	0.4	0.2
PC 60 BBE 12	0.2	0.05

**Table 9:** Physico-mechanical properties of composite polymeric films (PC) according to the content of birch bark extract (BBE) and the use of ultrasonic treatment (US).

Polymer Composition	Breaking Stress, MPa		Elongation at Break, %	
	with US	without US	with US	without US
PC 20 BBE 0	11	9.7	160	90
PC 40 BBE 0	9.1	7.8	58	19
PC 60 BBE 0	7.1	6.3	25	9
PC 20 BBE 2	10.5	9.2	158	90
PC 40 BBE 2	8.8	7.1	56	15
PC 60 BBE 2	6.7	5.9	24	8
PC 20 BBE 5	10.1	8.5	155	86
PC 40 BBE 5	8.1	6.7	42	13
PC 60 BBE 5	6.3	5.4	24	8
PC 20 BBE 8	9.7	7.9	140	67
PC 40 BBE 8	6.9	5.9	36	12
PC 60 BBE 8	5.9	4.6	10	6
PC 20 BBE 12	9.2	7.3	114	60
PC 40 BBE 12	6.7	5.4	33	12
PC 60 BBE 12	5.3	4.3	9	5

**Table 10:** Change in relative elongation at break of composite polymeric materials (PC) containing birch bark extract (BBE), received with (with US) and without the use of ultrasonic treatment (without US).

Polymer Composition	Change in Elongation at Break after Composting for 6 months, %	
	with US	without US
PC 20 BBE 0	20–23	16–18
PC 40 BBE 0	39–43	28–32
PC 60 BBE 0	78–81	67–69
PC 20 BBE 8	17–19	13–15
PC 40 BBE 8	32–35	24–28
PC 60 BBE 8	60–68	46–54
